# Structure and Immunocytochemical Analysis of Tracheoid Idioblasts in *Nepenthes* Pitchers

**DOI:** 10.3390/ijms27104223

**Published:** 2026-05-09

**Authors:** Bartosz J. Płachno, Małgorzata Kapusta, Marcin Feldo, Piotr Stolarczyk, Piotr Świątek

**Affiliations:** 1 Institute of Botany, Faculty of Biology, Jagiellonian University, 9 Gronostajowa St., 30-387 Kraków, Poland; 2 Bioimaging Laboratory, Faculty of Biology, University of Gdańsk, 59 Wita Stwosza St., 80-308 Gdańsk, Poland; malgorzata.kapusta@ug.edu.pl; 3 Department of Vascular Surgery and Angiology, Medical University of Lublin, 16 Staszica St., 20-081 Lublin, Poland; martinf@interia.pl; 4 Department of Botany, Physiology and Plant Protection, Faculty of Biotechnology and Horticulture, University of Agriculture in Kraków, 29 Listopada 54 Ave., 31-425 Kraków, Poland; 5 Institute of Biology, Biotechnology and Environmental Protection, Faculty of Natural Sciences, University of Silesia in Katowice, 9 Bankowa St., 40-007 Katowice, Poland; piotr.swiatek@us.edu.pl

**Keywords:** carnivorous plants, pitcher plants, *Nepenthes*, idioblasts, cell wall, secondary cell wall, cell wall microdomains, hemicelluloses, pectic homogalacturonan, xyloglucan, xylan

## Abstract

Tracheoid idioblasts in *Nepenthes* are anatomically specialized cells that differ distinctly from surrounding tissues in morphology, wall structure, and staining properties. Their presence has been documented in both vegetative organs, such as roots and stems, and in highly modified carnivorous leaves that form pitchers. We tested the hypothesis that if tracheoid idioblasts function to reinforce the mechanical strength of *Nepenthes* pitchers or to protect them from animal damage, they exhibit a secondary cell wall composition comparable to that of sclerenchyma or xylem cells, particularly with respect to its lignin and hemicellulose components. We assessed the localization of cell wall components in gland cell walls using histochemical tests, immunolabeling, and confocal microscopy. Light and scanning electron microscopy were used to reveal the tracheoid idioblast structure. Two species were examined: *Nepenthes albomarginata* T.Lobb ex Lindl. and *Nepenthes bicalcarata* Hook. f. In both species, giant tracheoid idioblasts with helical bands of secondary wall material were found throughout the pitchers. Negative phloroglucinol, fuchsin, and safranin staining test results demonstrated the absence of lignins in the tracheoid idioblast secondary cell walls. A histochemical test showed that the wall thickenings of the tracheoid idioblasts contained polysaccharides and cellulose and were rich in unsubstituted or low-substituted xylans, resembling the secondary cell walls of sclerenchyma and xylem cells. Our results suggest that tracheoid idioblasts with a helical secondary wall that is rich in xylans but not lignified most likely function as elastic reinforcing elements that increase the mechanical integrity of the organ while maintaining its flexibility and ability to undergo reversible deformation. Furthermore, tracheoid idioblasts may provide defense against herbivory.

## 1. Introduction

The term “idioblast” was proposed by Sachs [1] in the 19th century. Idioblasts are specialized plant cells that differ markedly in structure, content, or function from surrounding cells within the same tissue. Unlike neighboring cells, idioblasts are morphologically and physiologically distinct and are often characterized by specific wall modifications, intracellular inclusions (e.g., crystals, oils, tannins), or secondary wall thickenings. They may occur singly or in small groups and are typically associated with protective, secretory, mechanical, or storage functions [2,3,4,5].

Foster [2] distinguished three main types of idioblasts: excretory, tracheoid, and sclerenchymatous. Excretory (secretory) idioblasts, including oil cells, tannin cells, myrosin cells, mucilage cells, and crystal idioblasts, are widespread in plants and have been investigated intensively in numerous studies, e.g., References [6,7,8,9,10,11,12,13,14,15]. This sustained interest also stems from the fact that idioblasts may contain oils, secondary metabolites, or other substances of pharmaceutical or industrial importance [16,17,18].

Tracheoid idioblasts (also called tracheoids or helical idioblasts) resemble tracheids in that they possess spirally thickened or pitted secondary cell walls but lack typical tracheary elements, such as the perforation plates of vessel elements [2,19]. Research on tracheoid idioblasts in angiosperms has mainly focused on their occurrence in a taxonomic or phylogenetic context [20,21,22,23,24,25,26]. Unlike excretory idioblasts [15,27,28], detailed cytochemical analyses of the cell walls of tracheoid idioblasts of flowering plants remain scarce and have mostly involved the examination of secondary wall lignification, such as in Reference [20].

Tracheoid idioblasts were identified in *Nepenthes* organs as early as the 19th century. Mangin [29] reported their presence in stems, leaves (lamina), and pitchers. He described their development and noted that they are oriented in different directions, occur individually, and do not form groups. He stated that the spiral thickenings of the tracheoid idioblast cell walls are not lignified, as they did not stain with fuchsin. However, subsequent studies have produced divergent results. Owen and Lennon [30], who examined pitchers of *Nepenthes alata*, reported that these structures are lignified. According to Schwallier et al. [25], in the stems of many *Nepenthes* species, tracheoid idioblasts occur with either very thin lignified spiral walls or extremely thick lignified walls resembling fiber-sclereids.

Opinions also differ regarding the function of tracheoid idioblasts in *Nepenthes*. Mangin [29] stated that their function could not be clearly defined, although when present in large numbers, they might be considered supporting elements. Lloyd [31] supported the view that tracheoid idioblasts contribute to the mechanical reinforcement of pitcher walls. Still, he also agreed with Kny and Zimmermann [32] that they could be regarded as water reservoirs. Metcalfe and Chalk [33] likewise repeated the view that these tracheoid idioblasts (referred to as spiral elements) may serve a water-storage function. Carlquist [24] proposed that the helical wall bands of tracheoid idioblasts may retard foraging by forming an entangling structure when insects or other predators attempt to chew tissues containing these cells.

If tracheoid idioblasts function to reinforce the mechanical strength of *Nepenthes* pitchers or to protect them from animal damage, they should share structural similarities with sclerenchyma or xylem cells. Such similarities would be expected to be reflected in a comparable composition and organization of their secondary cell walls in terms of both the presence of lignin and other specific components of the cell wall. For example, it was shown that xylan epitopes recognized by LM10 and LM11 are ubiquitous components of secondary cell walls in vascular and mechanical plant tissues [34,35,36,37,38]. Thus, the aim of this study was to investigate the cell wall composition of tracheoid idioblasts from *Nepenthes* pitchers. Therefore, we performed immunolabeling experiments with a range of glycan-directed monoclonal antibodies [LM10 (unsubstituted or low-substituted xylans), LM11 (unsubstituted xylans), CCRC-M138 (xylan), LM25 (galactoxyloglucan), JIM5 (low methylesterified HGs), LM19 (low methylesterified HGs), JIM7 (highly esterified HGs), CCRC-M35 (rhamnogalacturonan-I backbone), LM2 (arabinogalactan protein), and JIM14 (arabinogalactan protein)] on *Nepenthes* tissues (*Nepenthes albomarginata* T.Lobb ex Lindl., and *Nepenthes bicalcarata* Hook. f.). We also performed phloroglucinol, fuchsin, and safranin staining tests demonstrate the occurrence or absence of lignins in the cell walls of tracheoid idioblasts. For our research, we selected two species that are highly interesting for their pitcher specializations. *Nepenthes albomarginata* has a tomentose band of tissue immediately beneath the peristome, which mimics lichens to attract termites [39,40]. *Nepenthes bicalcarata* has a mutualistic association with a species of swimming ant, *Camponotus schmitzi* Stärk [41,42,43], and produces unusual fang-like nectaries [44]. This species, unlike *N. albomarginata* [45], does not form a wax zone in the pitcher [46].

## 2. Results

### 2.1. Tracheoid Idioblast Occurrence and Structure

**Figure 1:**
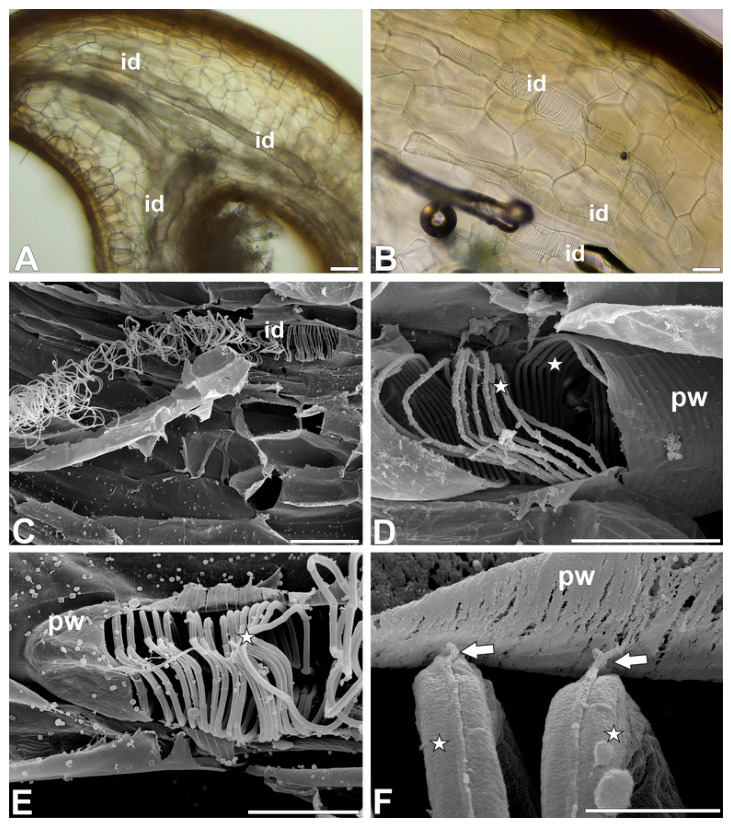
Occurrence and structure of *Nepenthes* tracheoid idioblasts. (**A**,**B**) Tracheoid idioblasts (id) in peristome of *Nepenthes albomarginata*, bar 100 µm and bar µm. (**C**) Damaged tracheoid idioblast (id) of *Nepenthes albomarginata*, bands of secondary wall material forming tangled threads, bar 100 µm. (**D**) Structure of tracheoid idioblast of *Nepenthes bicalcarata*; primary cell wall (pw), bands of secondary wall material (asterisk), bar 50 µm. (**E**,**F**) Structure of the tracheoid idioblast of *Nepenthes albomarginata*; primary cell wall (pw), bands of secondary wall material (asterisk). Note thin strands (arrow) that connect bands of secondary wall material with primary cell wall, bar 20 µm and bar 2 µm.

**Figure 2:**
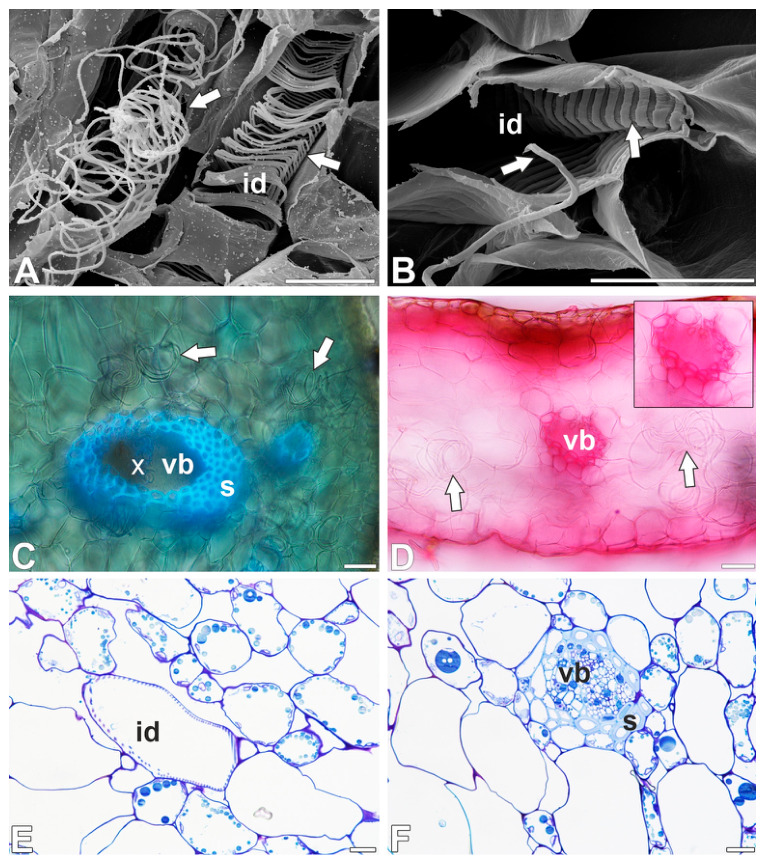
Tracheoid idioblast structure and histochemistry. (**A**,**B**) Two types of tracheoid idioblasts (id) in *Nepenthes albomarginata*, idioblast bands (arrow), bar 50 µm, and bar 40 µm. (**C**) Strong blue autofluorescence of the lignified cell walls of sclerenchyma cells (s) and wood vascular elements (x); note no such autofluorescence of idioblast bands of secondary wall material (arrow), bar 50 µm. (**D**) *Nepenthes albomarginata*. Result of phloroglucinol reaction for lignin, see positive result—pink staining of the cell walls of sclerenchyma cells of vascular bundle (vb) (enlarged fragment); no positive result in tracheoid idioblast bands of secondary wall material (arrow), bar 50 µm. (**E**,**F**) Sections through *Nepenthes bicalcarata* pitcher, stained with toluidine blue O. Idioblast (id), vascular bundle (vb), sclerenchyma (s), bar 25 µm, and bar 25 µm.

In both species, giant tracheoid idioblasts were commonly found in various parts of the pitchers (Figure 1A). The idioblasts were strongly elongated, resembling a spindle. The idioblasts usually occurred individually, surrounded by parenchyma cells, or two idioblasts occurred side by side (Figure 1B). However, three tracheoid idioblasts were also observed next to each other. A tracheoid idioblast may adhere to the vascular bundle, running parallel to it. However, the tracheoid idioblast were observed to be separated from the vascular tissues by sclerenchyma cells. During manual tissue cutting, tracheoid idioblasts were torn apart, and bands of secondary wall material were released, forming tangled threads (Figure 1C). These threads connected fragments of pitchers, making them difficult to separate. The primary cell wall was preserved in the tracheoid idioblast (Figure 1D–F). The tracheoid idioblasts had helical bands of secondary wall material. These bands are attached to the primary cell wall by thin strands (Figure 1F). The helices lie next to each other. In *Nepenthes albomarginata,* two types of tracheoid idioblasts were recorded: one with non-flattened, thin bands (more frequent) and another with flattened, wide bands (less frequent) (Figure 2A,B).

### 2.2. Histochemical Analysis of Tracheoid Idioblasts

**Figure 3:**
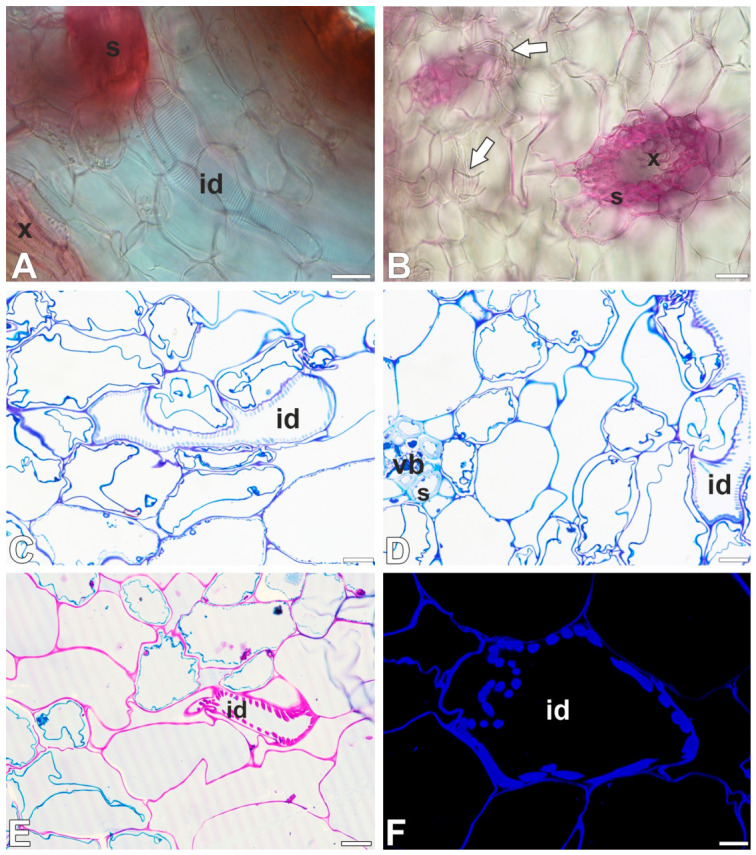
Tracheoid idioblast histochemistry. (**A**) *Nepenthes bicalcarata* Safranin staining, tracheoid idioblast secondary cell wall, xylem (x), sclerenchyma (s), bar 25 µm. (**B**) *Nepenthes albomarginata* fuchsin staining, tracheoid idioblast secondary cell wall (arrow), xylem (x), sclerenchyma (s), bar 50 µm. (**C**,**D**) Sections through *Nepenthes albomarginata* pitcher stained with methylene blue; idioblast (id), vascular bundle (vb), sclerenchyma (s), bar 25 µm, and bar 25 µm. (**E**) *Nepenthes albomarginata*. Result of periodic acid–Schiff reaction, note strong positive reaction in tracheoid idioblast cell wall (id), bar 25 µm. (**F**) *Nepenthes albomarginata* tracheoid idioblast (id) stained with Calcofluor White, which indicates presence of cellulose, bar 10 µm.

The wall thickenings of tracheoid idioblasts did not exhibit the strong autofluorescence that was observed in xylem vessels and sclerenchyma cells, suggesting the absence of lignin in the tracheoid idioblast cell walls (Figure 2C). In contrast, sclerenchyma and xylem vessel walls showed a characteristic pink coloration following phloroglucinol staining, confirming the presence of lignin (Figure 2D). The lack of a positive phloroglucinol reaction in tracheoid idioblasts further indicates that their secondary cell walls are not lignified (Figure 2D). Toluidine blue O staining was also used to assess lignification. Xylem vessels and sclerenchyma cell walls exhibited the characteristic light staining indicative of lignin’s presence. In contrast, the wall thickenings of tracheoid idioblasts did not display such staining (Figure 2E,F), supporting the conclusion that their secondary cell walls lack lignin. Safranin staining showed positive staining of sclerenchyma cell walls but negative staining of idioblast secondary cell walls (Figure 3A). A similar staining result was obtained using fuchsin (Figure 3B).

Methylene blue/azure II stained the wall thickenings of tracheoid idioblasts or pink-purple or blue, while the thickenings of the vessels were light blue in color (Figure 3C,D).

A positive periodic acid–Schiff reaction result indicated that the wall thickenings of tracheoid idioblasts contain polysaccharides (Figure 3E). The wall thickenings of tracheoid idioblasts were stained with Calcofluor White (Figure 3F).

### 2.3. Cell Wall Components

**Figure 4:**
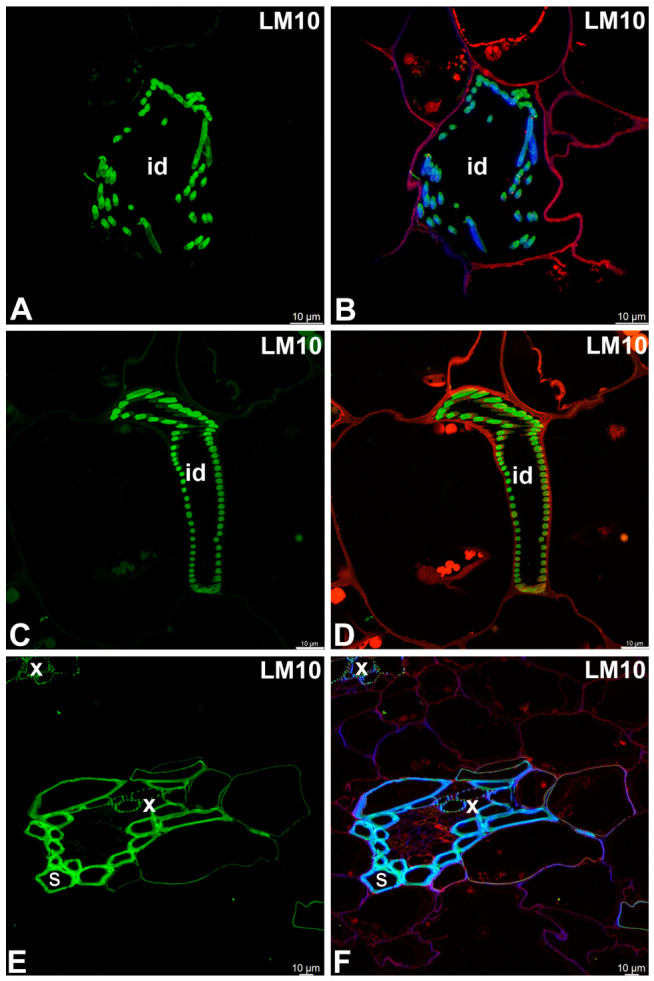
Unsubstituted or low-substituted xylans detected in the tracheoid idioblasts and vascular bundle of the *Nepenthes*, (intense green color—signal of antibody, red-brown color—autofluorescence, blue color—cellulose, staining with Calcofluor White). (**A**,**B**) Labeling with LM10 antibody, tracheoid idioblast in *Nepenthes albomarginata*; idioblast (id). (**C**,**D**) Labeling with LM10 antibody, tracheoid idioblast in *Nepenthes bicalcarata*; idioblast (id). (**E**,**F**) Labeling with LM10 antibody, vascular bundle in *Nepenthes albomarginata*; sclerenchyma cells (s) and tracheary elements of xylem (x). All bars 10 µm.

**Figure 5:**
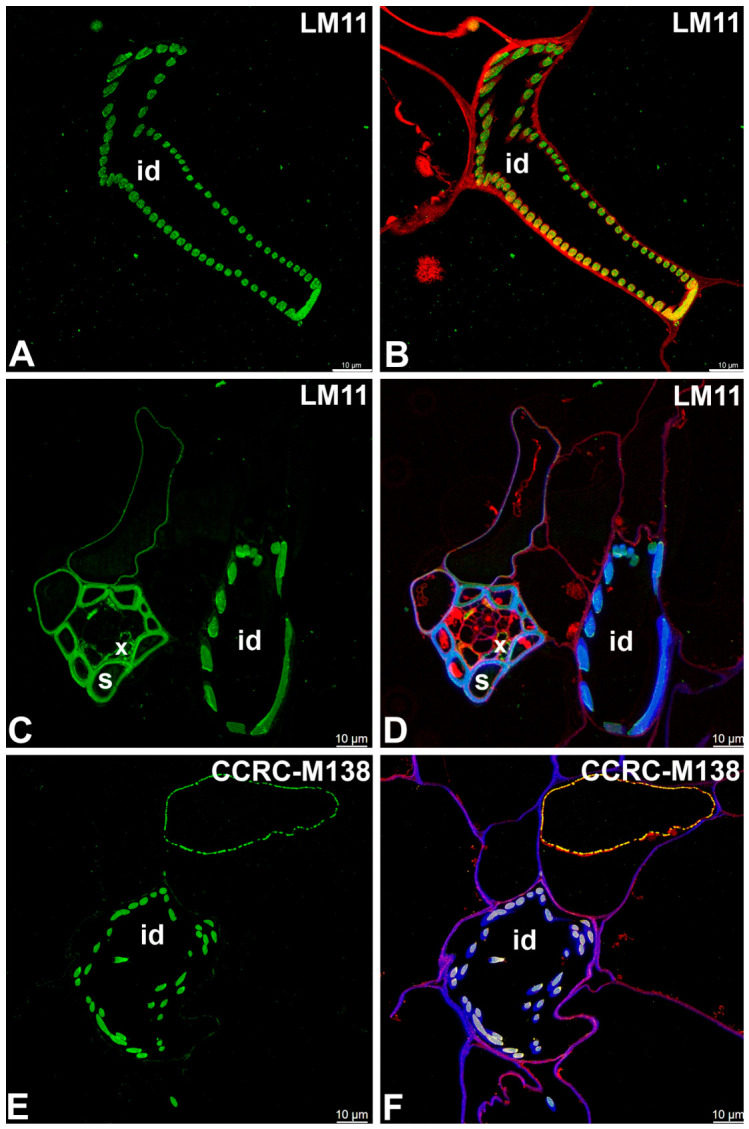
The unsubstituted xylans and glycan group of xylan-6 detected in the idioblasts and vascular bundle of the *Nepenthes*, (intense green color—signal of antibody, red-brown color—autofluorescence, blue color—cellulose, staining with Calcofluor White). (**A**,**B**) Labeling with LM11 antibody, tracheoid idioblast in *Nepenthes bicalcarata*; idioblast (id). (**C**,**D**) Labeling with LM11 antibody, tracheoid idioblast and vascular bundle in *Nepenthes albomarginata*; idioblast (id), sclerenchyma cells (s), and tracheary elements of xylem idioblast (x). (**E**,**F**) Labeling with CCRC-M138 antibody, tracheoid idioblast in *Nepenthes albomarginata*; idioblast (id). All bars 10 µm.

**Figure 6:**
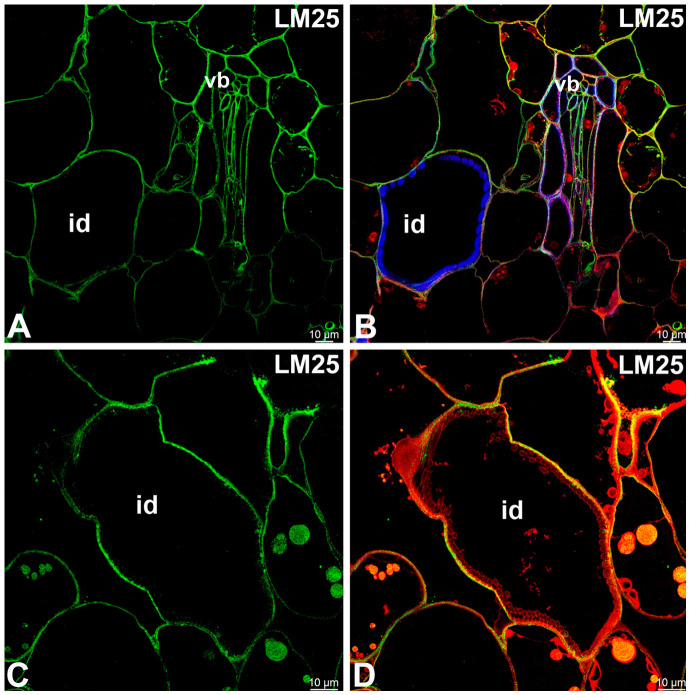
The galactoxyloglucan in the tracheoid idioblasts of the *Nepenthes*, (intense green color—signal of antibody, red-brown color—autofluorescence, blue color—cellulose, staining with Calcofluor White). (**A**,**B**) Labeling with LM25 antibody, tracheoid idioblast in *Nepenthes albomarginata*; idioblast (id), vascular bundle (vb). (**C**,**D**) Labeling with LM25 antibody, tracheoid idioblast in *Nepenthes bicalcarata*; idioblast (id). All bars 10 µm.

A monoclonal antibody to the unsubstituted or low-substituted xylans, LM10, bound strongly to the helical secondary wall thickenings of tracheoid idioblasts (Figure 4A–D). The LM10 epitope was detected in the cell walls of sclerenchyma cells and tracheal elements of the xylem (Figure 4E,F). The LM11 epitope (unsubstituted xylans) had a similar pattern of occurrence (Figure 5A–D) as LM10. Fluorescence labeling with CCRC-M138, which recognizes the glycan group of xylan-6, was also detected in the helical secondary wall thickenings of tracheoid idioblasts (Figure 5E,F). Galactoxyloglucan epitopes detected by LM25 were distributed only in the primary cell walls of tracheoid idioblasts (Figure 6A–D).

**Figure 7:**
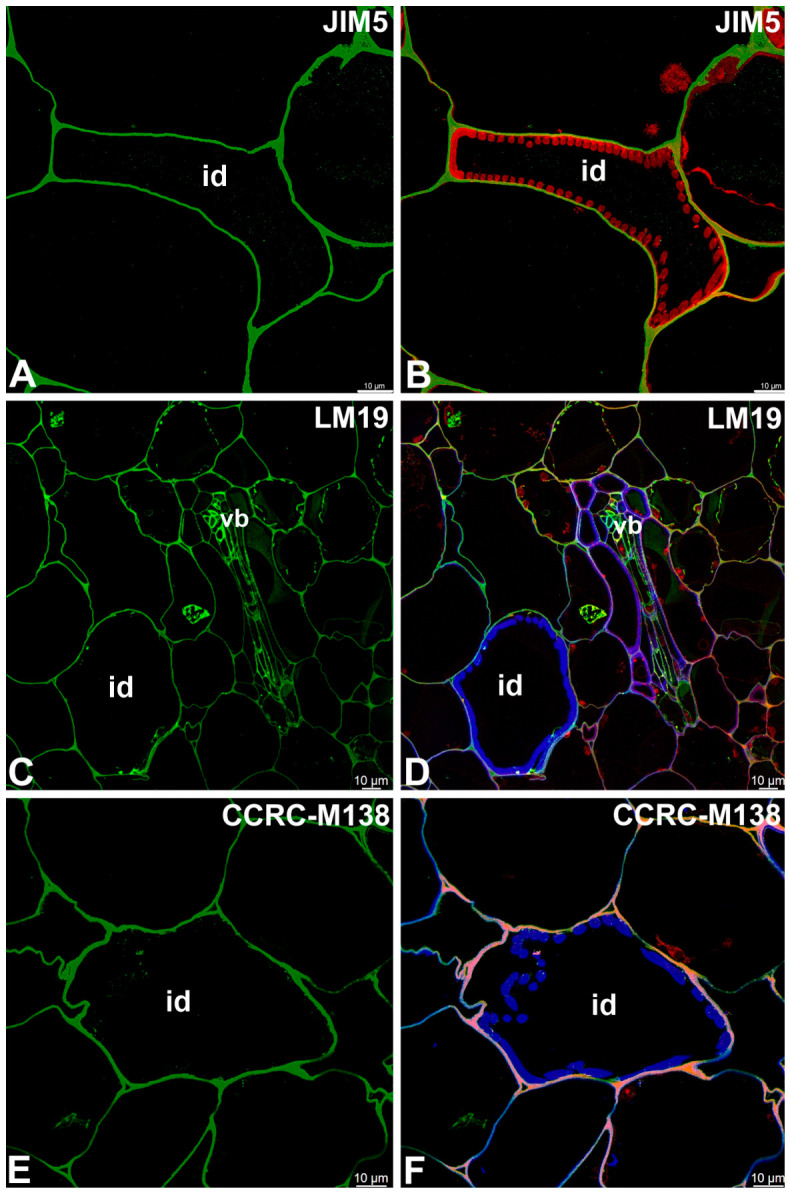
The pectic homogalacturonan (low-methylesterified and de-methylesterified HGs) detected in the tracheoid idioblasts of the *Nepenthes*, (intense green color—signal of antibody, red-brown color—autofluorescence, blue color—cellulose, staining with Calcofluor White). (**A**,**B**) Labeling with JIM5 antibody, tracheoid idioblast in *Nepenthes bicalcarata*; idioblast (id). (**C**,**D**) Labeling with CCRC-M38 antibody, tracheoid idioblast in *Nepenthes albomarginata*; idioblast (id), vascular bundle (vb). (**E**,**F**) Labeling with LM19 antibody, tracheoid idioblast in *Nepenthes albomarginata*; idioblast (id). All bars 10 µm.

**Figure 8:**
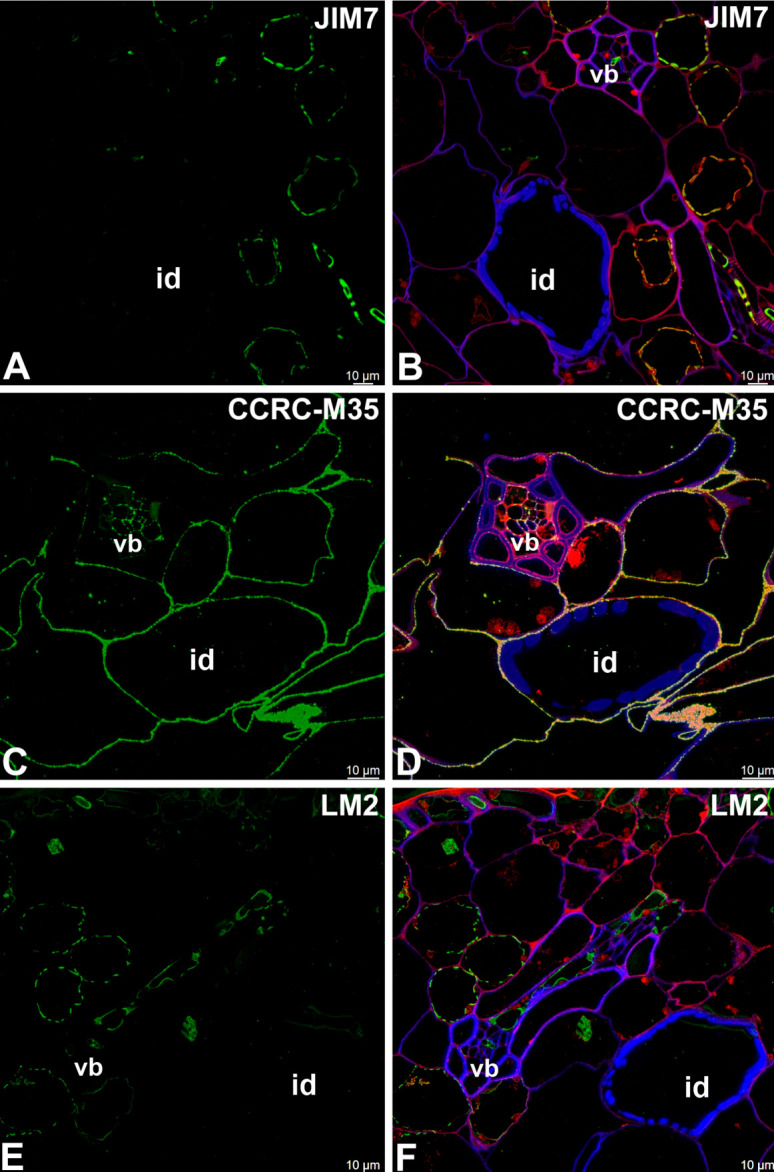
Cell wall components detected in the tracheoid idioblasts of the *Nepenthes*, (intense green color—signal of antibody, red-brown color—autofluorescence, blue color—cellulose, staining with Calcofluor White). (**A**,**B**) Labeling with JIM7 antibody (highly esterified HGs), tracheoid idioblast in *Nepenthes albomarginata*; idioblast (id), vascular bundle (vb). (**C**,**D**) Labeling with CCRC-M35 antibody (Rhamnogalacturonan-I backbone), tracheoid idioblast in *Nepenthes albomarginata*; idioblast (id), vascular bundle (vb). (**E**,**F**) Labeling with LM2 antibody (arabinogalactan proteins), tracheoid idioblast in *Nepenthes albomarginata*; idioblast (id), vascular bundle (vb). All bars 10 µm.

JIM5 and LM19 (low methylesterified Homogalcturonan [HG]) and CCRC-M38 (de-esterified α-1,4 linked Homogalcturonan) were observed in the primary cell wall of tracheoid idioblasts; however, they were not observed not in the helical secondary wall thickenings (Figure 7A–F). The highly esterified HGs (detected by JIM7) were not detected in either the primary or secondary cell walls of tracheoid idioblasts (Figure 8A,B).

CCRC-M35 (Rhamnogalacturonan-I backbone) occurred in the primary cell wall of tracheoid idioblasts but was not detected in the helical secondary wall thickenings (Figure 8C,D). The arabinogalactan proteins (LM2 and JIM14 epitopes) were lacking in helical cell wall thickenings (Figure 8E,F).

## 3. Discussion

### 3.1. Non-Lignified Tracheoid Idioblast Secondary Cell Walls?

The most popular test for lignin is the reaction involving phloroglucinol and acid. We obtained a negative result for this staining method in idioblasts. However, some researchers postulate that impregnation of the walls with phenolic compounds may hinder the detection of lignin (see the discussion in [47]), and various secondary compounds have been found in *Nepenthes* [48,49,50]. However, unlike ferns, *Nepenthes* lacks the characteristic dark impregnation of cell walls, and phenolic compounds are stored in vacuoles. Furthermore, both safranin and fuchsin staining revealed lignin in the cell walls of sclerenchyma cells, whereas tracheoid idioblasts lacked it. The difference also concerned the autofluorescence of cell walls. The lignified cell walls of xylem and sclerenchyma cells showed strong (white-blue) autofluorescence, unlike the secondary walls of tracheoid idioblasts. Thus, our research (based on histochemical tests, cell wall autofluorescence, and comparison with sclerenchyma cells) indicates that the secondary cell walls of tracheoid idioblasts in pitchers of the analyzed species lack lignification. In future studies, in addition to cytochemical staining, it is worth using other techniques such as Raman spectroscopy or FTIR analysis.

These results are consistent with the observations of Mangin [29], who also found no lignin in spiral thickenings based on a negative fuchsin staining result (in *Nepenthes phyllamphora* = *Nepenthes mirabilis*). Owen and Lennon [30] proposed that these structures are lignified in *Nepenthes alata* because toluidine blue stained the thickenings a light blue. On the other hand, Schwallier et al. [25] reported lignification of tracheoid idioblast walls in various *Nepenthes* species. However, these authors studied tracheoid idioblasts from stems and not pitchers. Therefore, differences caused by the location of tracheoid idioblasts (e.g., the need for mechanical reinforcement of a given organ) cannot be ruled out, especially since Osunkoya et al. [51] found differences in lignin content between leaf lamina and pitchers in several *Nepenthes* species. These authors found that pitchers contained less lignin than leaf laminae despite their complex, fibrous appearance. Additionally, inter-species differences cannot be ruled out.

Regarding tracheoid idioblasts and similar cells with spiral thickenings, there are various reports on the lignification of the secondary cell wall. Such cells are common in orchids. Olatunji and Nengim [23] reported that most tracheoid idioblast in orchids were lignified. However, they also mentioned that in certain cases, the lignification may be slight. Also, the secondary walls of the orchid velamen (which arise from cells of the rhizodermis) are lignified [52]. The tracheoid cells, forming velamen-like tissues (pseudovelamen, derived from root cortex cells) in the root cortex of many epiphytic orchid species, have lignified annular thickenings [53]. However, in *Salicornia europaea* (Chenopodiaceae) leaves, the secondary cell walls of tracheoid idioblasts did not stain with iodine green, indicating a lack of lignification [54]. It was extremely interesting to note that, among numerous fern species (family Aspleniaceae), the helical cell wall thickenings were non-lignified [47]. These authors emphasize that unlike lignified secondary cell walls, which have been studied extensively, non-lignified secondary cell walls have received little attention.

### 3.2. Presence of Cell Wall Components in Tracheoid Idioblasts

Calcofluor white staining of the secondary cell walls of tracheoid idioblasts indicated an abundance of cellulose. We observed the CCRC-M138 epitope (the glycan group of xylan-6) in the secondary cell walls of tracheoid idioblasts. We identified LM10 and LM11 epitopes in the secondary cell walls of tracheoid idioblasts, xylem, and sclerenchyma. Unsubstituted xylans are abundant components of the secondary cell walls associated with the vascular tissues and fibers in dicotyledons [34,36]. Carafa et al. [35] showed that these epitopes are widespread in tracheary elements in tracheophytes. The explanation for the evolutionary success of plants that use xylans is that xylans interact intimately with cellulose microfibrils, forming a hemicellulose–cellulose network that stabilizes the wall architecture. Solid-state NMR studies show that xylan can adopt a specific conformation (twofold helical screw) that enables hydrogen bonding to the hydrophilic surfaces of cellulose, effectively extending microfibril arrays and modifying their association with other polymers [55]. Through their interactions with cellulose, xylans contribute to the mechanical strength and flexibility of cell walls. Studies indicate that xylans influence the extensibility of cell walls, thereby affecting both stiffness and deformation resistance, depending on wall composition [56].

Leroux et al. [47] examined helical cell wall thickenings in ferns with respect to wall components. These authors did not find the LM11 epitope in the helical cell wall thickenings of ferns. However, they detected cellulose, homogalacturonans, and xyloglucan. We also detected cellulose and general polysaccharides (positive periodic acid–Schiff reaction) in helical cell wall thickenings of *Nepenthes* tracheoid idioblasts, but we did not find low methylesterified homogalacturonans (JIM5, LM19 epitopes), de-esterified α-1,4-linked homogalacturonans (CCRC-M38 epitope), galactoxyloglucans (LM25 epitope), or rhamnogalacturonan (CCRC-M35) in the helical cell wall thickenings of *Nepenthes* tracheoid idioblasts. There are, therefore, differences between the primary cell wall and the secondary cell wall. We also showed that arabinogalactan proteins (LM2 and JIM14 epitopes) were lacking in these helical cell wall thickenings.

### 3.3. Possible Function(s) of Tracheoid Idioblasts

Cell walls that are not lignified but are rich in xylans are more elastic, exhibit greater elastic and partially plastic deformation capacity, and are more easily compressed [55,57,58]. Thus, we propose that tracheoid idioblasts with a helical secondary wall rich in xylans but not lignified most likely function as elastic reinforcing elements that increase the mechanical integrity of the organ while maintaining its flexibility and ability to undergo reversible deformation. These are not conductive or typically supportive elements in the sclerenchymatic sense; rather, they are intermediate structures—stabilizing and cushioning. In addition to tracheoid idioblasts, vascular bundles surrounded by sclerenchyma fibers perform a mechanical function in pitchers. This is particularly important as these fibers have lignified walls. We propose that both systems, tracheoid idioblasts and vascular bundles, which work together, are important in pitcher mechanics.

Tracheoid idioblasts in *Nepenthes* pitchers may also serve another purpose. We agree with Carlquist [24] that the tendency of tracheoid idioblast cell wall helis to disintegrate into a tangled mass when physically disturbed makes it difficult for herbivores to eat the pitchers. However, this strategy is not sufficient in young *Nepenthes* leaves, which insects destroy. For example, Merbach et al. [59] observed that weevils caused severe damage to developing leaf parts, especially young pitchers. We assume that in such developing leaves, tracheoid idioblasts are not fully developed (see [29]), but this remains to be confirmed. Osunkoya et al. [51] showed that *Nepenthes* species differ in terms of pitcher longevity. According to these authors, the lifespan of pitchers ranges from less than a month (*N. mirabilis*) to six months (*N. bicalcarata*). It would be interesting to see how idioblasts change over the life of a pitcher. For example, do changes occur in the secondary walls of idioblasts after a few months?

It is worth asking whether conditions within the pitcher, including environmental high humidity and microbial activity, could contribute to the development of the unusual xylan-rich secondary walls as an adaptation against biological degradation? If these cells are involved in mechanical or defensive functions, the habitat may play a regulatory role in their formation. To prove this hypothesis, appropriate experiments must be planned.

It should be noted that despite similar trap structures, *Nepenthes* exhibit enormous diversity in terms of pitcher size and geometry, peristome shape and structure, and feeding specialization [60,61,62,63,64]. Therefore, further research should focus on whether there is a relationship between the structural specialization of pitchers and the occurrence and chemical structure of tracheoid idioblasts.

## 4. Materials and Methods

### 4.1. Plant Material

Mature *Nepenthes bicalcarata* Hook. f. plants were grown in the warm greenhouse of the Faculty of Biology, University of Gdansk. *Nepenthes albomarginata* T.Lobb ex Lindl. pitchers were taken from the collection of the first author. Mature *Nepenthes albomarginata* plants were grown in a humid terrarium at room temperature. A mixture of acidic peat and sand was used as the substrate. Fully developed, open pitchers were selected for the study.

### 4.2. Histological and Immunochemical Analysis

The traps were cut into small fragments and fixed as described by Płachno et al. [65]. For analysis of the occurrence of the major cell wall polysaccharides and glycoproteins, the plant material was dehydrated with acetone and embedded in LR White Acrylic Resin (Merck Life Science Sp.z.o.o., an affiliate of Merck KGaA, Darmstadt, Germany). Sections were cut on a Leica Ultracut UCT ultramicrotome (0.7 µm thick). The rehydrated sections in PBS buffer were blocked with 1% bovine serum albumin (BSA, Sigma-Aldrich, St. Louis, MI, USA) in a PBS buffer and incubated with the following primary antibodies overnight at 4 °C: LM10 (unsubstituted or low-substituted xylans), LM11 (unsubstituted xylans), CCRC-M138 (xylan), LM25 (galactoxyloglucan), JIM5 (low methylesterified HGs), LM19 (low methylesterified HGs), JIM7 (highly esterified HGs), CCRC-M35 (rhamnogalacturonan-I backbone), LM2 (arabinogalactan protein), and JIM14 (arabinogalactan protein) [66,67]. All primary antibodies were used at a 1:20 dilution. They were purchased from Plant Probes, UK (rat monoclonal antibodies) and Agrisera, Sweden (mouse monoclonal antibodies). Secondary antibodies: goat anti-rat secondary or anti-mouse antibody conjugated with FITC (fluorescein isothiocyanate) [excitation wavelength (λEx): ~495 nm, emission wavelength (λEm): ~519 nm] or Alexa Fluor 488 [excitation wavelength (λEx): 495 nm, emission wavelength (λEm): 519 nm], respectively, were purchased from Abcam (Cambridge, UK). The samples were then cover-slipped using a Mowiol mounting medium: a mixture of Mowiol ^®^4-88 (Sigma-Aldrich) and glycerol for fluorescence microscopy (Merck, Warsaw, Poland) with the addition of 2.5% DABCO (Carl Roth GmbH + Co. KG, Karlsruhe, Germany). They were viewed using a Leica STELLARIS 5 WLL confocal microscope with Lightning module deconvolution. Negative controls were created by omitting the primary antibody step, which caused no fluorescence signal in any of the control frames for any stained slides (Figure S1).

Semi-thin sections (0.7–1.0 µm thick) were prepared for light microscopy (LM) and stained for general histology using aqueous methylene blue/azure II (MB/AII).

### 4.3. Histochemical Analysis

Fresh hand sections (unstained or treated with alum carmine and iodine green) and unstained plastic sections were examined for autofluorescence of modified cell walls. For lignification detection, we used phloroglucinol staining [68], toluidine blue O [69], safranin and fuchsin [68]. Documentation was performed with a Nikon Eclipse E400 microscope (Nikon Europe B.V. Stroombaan 14, 1181 VX, Amstelveen, The Netherlands). The PAS reaction (periodic acid-Schiff reaction) was used to detect the polysaccharides [70]. Crystalline cellulose was also labeled using Calcofluor White Stain (Merck Life Science Sp.z.o.o., an affiliate of Merck KGaA, Darmstadt, Germany). Sections were viewed using a Leica STELLARIS 5 WLL confocal microscope or a Leica DM6000B microscope equipped with a DAPI (Ex/Em = 350/450 nm wavelength; exposure time, 347.136 ms with gain = 1) and a Rhodamine filter (Ex/Em = 546/585 nm wavelength; exposure time, 661.156 ms with gain = 1.9).

### 4.4. Scanning Electron Microscopy

To prepare for scanning electron microscopy (SEM), the traps were cut into small fragments and treated with sodium hypochlorite (10% or 20% aqueous solution of Ace Classic, Fater Central Europe, Bucaresti, Romania) and later washed in water, transferred to ethanol, and then transferred to acetone and dried using supercritical CO_2_. The material was then sputter-coated with gold and examined using a Hitachi S-4700 scanning electron microscope (Tokyo, Japan), which is housed at the Institute of Geological Sciences, Jagiellonian University, Kraków, Poland, or a Hitachi UHR FE-SEM SU 8010 microscope, which is housed at the Institute of Biology, Biotechnology and Environmental Protection, Faculty of Natural Sciences University of Silesia in Katowice.

## 5. Conclusions

To date, the cell walls of *Nepenthes* tracheoid idioblasts have not been analyzed in detail. Our findings regarding the presence or absence of specific components of these cell walls are entirely new to science.

The secondary cell walls of tracheoid idioblasts in *Nepenthes* pitchers appear to be non-lignified in both examined species. Immunolabelling revealed the presence of LM10, LM11, and CCRC-M138 epitopes, indicating that these secondary walls contain substantial amounts of xylans. The presence of xylans together with cellulose suggests a hemicellulose–cellulose network typical of secondary cell walls. Such networks can stabilize the wall architecture through hydrogen bonding between xylan and cellulose microfibrils, thereby influencing wall stiffness, extensibility, and mechanical resilience.

Mechanical reinforcement of the pitchers likely results from the interaction of two main structural elements. Tracheoid idioblasts with flexible, xylan-rich walls may function together with lignified sclerenchyma surrounding the vascular bundles, forming a mechanical system in which sclerenchyma provides rigid structural support. In contrast, tracheoid idioblasts contribute elasticity and resistance to deformation. The tendency of the helical wall thickenings to disintegrate into tangled masses when mechanically disturbed may additionally reduce tissue palatability or make the pitchers more difficult for herbivores to consume.

Future studies should examine how the structure of tracheoid idioblasts changes during pitcher development and aging and whether the chemical composition and distribution of these cells correlate with the remarkable diversity of pitcher morphology and trapping strategies observed in *Nepenthes*.

## Data Availability

The original contributions presented in this study are included in the article/Supplementary Materials. Further inquiries can be directed to the corresponding author.

## Supplementary Materials

The following supporting information can be downloaded at https://www.mdpi.com/article/10.3390/ijms27104223/s1.
