# Supplementary material for: Structure and Immunocytochemical Analysis of Tracheoid Idioblasts in Nepenthes Pitchers
Source: Int J Mol Sci. 2026 May 9;27(10):4223. doi: 10.3390/ijms27104223 (PMC13207644; doi:10.3390/ijms27104223)
Supplement: Supplementary file 1 [file ijms-27-04223-s001.zip › ijms-4281749-supplementary.pdf]

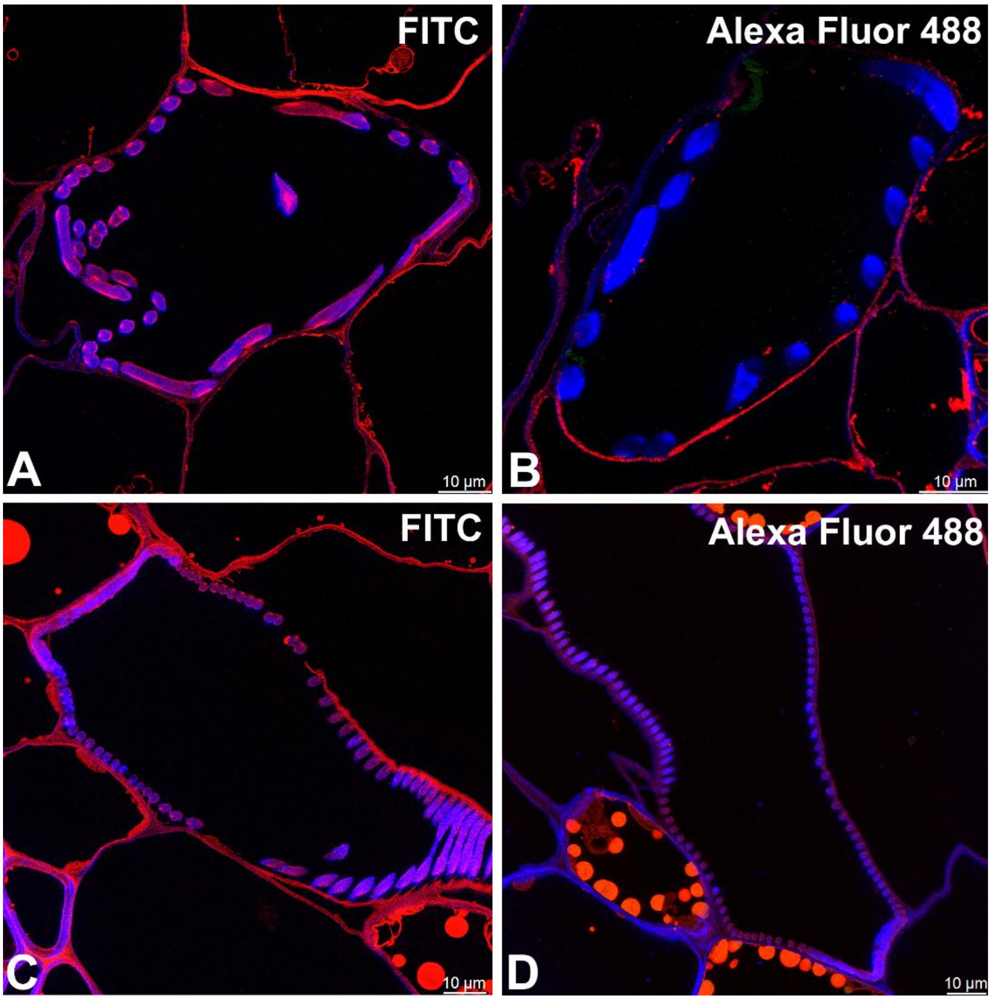

Figure S1. Control reactions for immunolabellings (green – antibody signal, red-brown – autofluorescence, blue – cellulose stained with Calcofluor White). **(A-B)** Negative controls for immunofluorescence using FITC-conjugated secondary antibody and Alexa Fluor 488-conjugated secondary antibody, without primary antibody, in *Nepenthes albomarginata.* **(C-D)** Negative controls for immunofluorescence using FITC-conjugated secondary antibody and Alexa Fluor 488-conjugated secondary antibody, without primary antibody, in *Nepenthes bilcalcarata*. All scale bars = 10 µm.
